# Optimized M‐SHAKE Constraint Implementations for GPU‐Accelerated Molecular Dynamics: Balancing Precision and Performance Across Architectures

**DOI:** 10.1002/jcc.70492

**Published:** 2026-09-17

**Authors:** Jaewoon Jung, Diego Ugarte La Torre, Chigusa Kobayashi, Katsuhisa Ozaki, Yuji Sugita

**Affiliations:** ^1^ Computational Biophysics Team RIKEN Center for Computational Science Kobe Japan; ^2^ Theoretical Molecular Science Laboratory RIKEN Pioneering Research Institute Wako Japan; ^3^ Department of Mathematical Science Shibaura Institute of Technology Saitama Japan; ^4^ Department of Physics University of Tokyo Tokyo Japan

## Abstract

In molecular dynamics (MD) simulations, enforcing holonomic constraints on specific interatomic distances is crucial to extend the integration time step while maintaining numerical stability. While double‐precision arithmetic has traditionally been used to ensure numerical accuracy, there is a growing demand for single‐ and mixed‐precision calculations as GPU‐accelerated MD simulations become increasingly widespread. In this study, we present improvements to the constraint solvers through an analytical treatment for bonds involving a single hydrogen atom ($XH_{1}$ groups). For more complex groups with multiple hydrogens ($XH_{2}$ and $XH_{3}$), we introduce two precision‐level implementation strategies: (1) a mixed‐precision approach, where variables are generally described in single‐precision while temporary coordinate updates are handled in double‐precision and (2) a pairwise arithmetic approach, where temporary coordinate variables are represented as pairs of single‐precision values to reduce rounding errors. These implementation strategies reduce velocity‐update errors compared with schemes using single precision for all variables. Energy drift analysis in microcanonical simulations confirms that these strategies maintain accuracy comparable to full double‐precision implementations. Performance benchmarks on GPUs with limited FP64 throughput (RTX 3080 and A6000 Ada) show that our proposed implementation strategies substantially reduce runtime compared with full double‐precision calculations. Conversely, on architectures with balanced FP32 and FP64 performance, such as CPUs or H100 GPUs, full double‐precision arithmetic remains the preferred choice for the constraint kernels evaluated in this study. Overall, these findings demonstrate that the proposed strategies provide an effective balance between numerical accuracy and computational performance on GPUs with limited FP64 throughput.

## Graphical abstract

Mixed‐precision (MP) and pairwise single‐precision (PW‐SP) implementations of the M‐SHAKE constraint solver reduce numerical errors and energy drift relative to pure single precision. On GPUs with limited FP64 throughput, both PW‐SP and MP outperform full double precision, with PW‐SP providing the largest speedup.

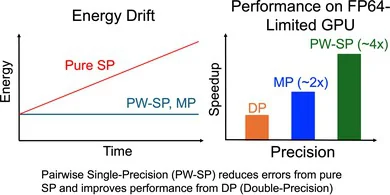

## 1 Introduction

Molecular Dynamics (MD) simulations have become indispensable for investigating biomolecular systems, offering crucial insights into biological phenomena at the atomic level [1]. One of the central challenges in MD is increasing the simulation time step without compromising numerical accuracy, which is widely achieved by applying constraints to fix fast vibrational modes, such as bonds involving hydrogen atoms [2, 3, 4, 5, 6]. Various constraint algorithms, including SHAKE [2], RATTLE [3], M‐SHAKE [6], LINCS [5], and SETTLE [4] have been developed to enforce these geometric conditions [5]. To achieve tight constraint tolerances and maintain long‐term numerical stability, these algorithms traditionally require high‐precision coordinate and velocity corrections.

While modern MD simulations increasingly rely on GPU acceleration to enhance throughput, the choice of floating‐point precision introduces a critical trade‐off. Achieving strict tolerances with conventional double‐precision (FP64) arithmetic introduces a severe performance penalty on many GPU architectures—particularly those with limited FP64 throughput—where FP64 compute units are scarce compared to single‐precision (FP32) units. Conversely, moving entirely to FP32 arithmetic introduces noticeable velocity‐update errors and accelerates energy drift, compromising the reliability of the trajectory. Therefore, a critical challenge is to retain high numerical accuracy in constraint enforcement while reducing the expensive reliance on full FP64 operations.

In this work, we develop optimized M‐SHAKE implementations that preserve strict numerical accuracy while improving computational efficiency on GPU architectures. Specifically, our contribution consists of three distinct components: (1) an analytical, noniterative treatment for bonds involving a single hydrogen atom ($XH_{1}$ groups) to eliminate iterative constraint correction for these bonds, (2) a mixed‐precision implementation strategy for handling more complex multihydrogen groups ($XH_{2}$ and $XH_{3}$), in which FP64 arithmetic is restricted to temporary coordinate updates, and (3) a pairwise arithmetic strategy that replaces selected FP64 coordinate‐update operations with pairs of FP32 values to reduce rounding errors [7]. We evaluate these approaches on CPUs and GPUs with different FP64 capabilities to characterize their specific accuracy‐performance trade‐offs.

## 2 Methods

### 2.1 Molecular Dynamics Integration

The numerical integration of Newton's equations of motion determines the accuracy and stability of MD simulations. Although several integration schemes are available, this work focuses on the velocity Verlet algorithm [8], which synchronizes positions and velocities at the same time points and therefore provides a convenient framework for applying both coordinate and velocity constraints. The update procedure consists of (1) advancing the velocities to the half step (Equation (1)), (2) updating the coordinates to the full step (Equation (2)), (3) applying coordinate constraints and the corresponding velocity correction, (4) evaluating the forces at the constrained coordinates, (5) advancing the velocities to the full step (Equation (3)), and (6) applying the velocity constraints to obtain the final constrained velocities.

(1) $$v_{i}^{*}\left(t+\frac{∆t}{2}\right)=v_{i}\left(t\right)+\frac{∆t}{2m_{i}}F_{i}\left(t\right)$$

(2) $$r_{i}^{*}\left(t+∆t\right)=r_{i}\left(t\right)+∆tv_{i}^{*}\left(t+\frac{∆t}{2}\right)$$

(3)

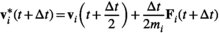




### 2.2 Constraint Scheme in Molecular Dynamics

#### 2.2.1 M‐SHAKE Constraint Scheme

For an $XH_{n}$ group (where X denotes a heavy atom, and H denotes hydrogen, with n=1,2,3), we define the unconstrained coordinates at t+∆t as $r_{0}^{*}\left(t+∆t\right)$ for the heavy atom and $r_{\mathrm{hi}}^{*}\left(t+∆t\right)$ for each hydrogen atom i=1,…,n, while the constrained coordinates at time t are denoted as $r_{0}\left(t\right)$ and $r_{\mathrm{hi}}\left(t\right)$. We then define the corresponding bond vectors as $r_{0i}^{*}=r_{0}^{*}\left(t+∆t\right)-r_{\mathrm{hi}}^{*}\left(t+∆t\right)$ and $r_{0i}=r_{0}\left(t\right)-r_{\mathrm{hi}}\left(t\right)$. For $XH_{2}$ and $XH_{3}$ groups, M‐SHAKE constructs a constraint matrix based on these bond vectors, which are formulated as:

(4) $$A_{XH_{2}}=\left[\begin{array}{cc} \left(\frac{1}{m_{X}}+\frac{1}{m_{H}}\right)r_{01}^{*}\cdot r_{01} & \frac{1}{m_{X}}r_{01}^{*}\cdot r_{02}\\ \frac{1}{m_{X}}r_{02}^{*}\cdot r_{01} & \left(\frac{1}{m_{X}}+\frac{1}{m_{H}}\right)r_{02}^{*}\cdot r_{02} \end{array}\right]$$

(5) $$A_{XH_{3}}=\left[\begin{array}{ccc} \left(\frac{1}{m_{X}}+\frac{1}{m_{H}}\right)r_{01}^{*}\cdot r_{01} & \frac{1}{m_{X}}r_{01}^{*}\cdot r_{02} & \frac{1}{m_{X}}r_{01}^{*}\cdot r_{03}\\ \frac{1}{m_{X}}r_{02}^{*}\cdot r_{01} & \left(\frac{1}{m_{X}}+\frac{1}{m_{H}}\right)r_{02}^{*}\cdot r_{02} & \frac{1}{m_{X}}r_{02}^{*}\cdot r_{03}\\ \frac{1}{m_{X}}r_{03}^{*}\cdot r_{01} & \frac{1}{m_{X}}r_{03}^{*}\cdot r_{02} & \left(\frac{1}{m_{X}}+\frac{1}{m_{H}}\right)r_{03}^{*}\cdot r_{03} \end{array}\right]$$

Here, $m_{X}$ and $m_{H}$ are the masses of the heavy and hydrogen atoms, respectively. The vector of Lagrange multipliers $\left(\lambda \right)$ is then obtained by inverting this matrix and multiplying it by the vector of squared constraint deviations $\left(\delta_{i}={\left|r_{0i}^{*}\right|}^{2}-d_{0i}^{2}\right)$:

(6) $$\left[\begin{array}{c} \lambda_{1}\\ \vdots \\ \lambda_{n} \end{array}\right]=A_{XH_{n}}^{-1}\left[\begin{array}{c} \delta_{1}\\ \vdots \\ \delta_{n} \end{array}\right]$$

Using these multipliers, the coordinates and half‐step velocities are updated simultaneously to satisfy the bond constraints:

(7) $$\begin{array}{l} r_{0}^{*}\left(t+\mathrm{\Delta t}\right)\to r_{0}^{*}\left(t+\mathrm{\Delta t}\right)-\frac{1}{2m_{X}}\sum_{i=1}^{n}\lambda_{i}r_{0i}\\ r_{\mathrm{hi}}^{*}\left(t+\mathrm{\Delta t}\right)\to r_{\mathrm{hi}}^{*}\left(t+\mathrm{\Delta t}\right)+\frac{1}{2m_{H}}\left(\lambda_{i}r_{0i}\right)\\ v_{0}^{*}\left(t+\frac{\mathrm{\Delta t}}{2}\right)\to v_{0}^{*}\left(t+\frac{\mathrm{\Delta t}}{2}\right)-\frac{1}{2m_{X}}\frac{1}{\mathrm{\Delta t}}\sum_{i=1}^{n}\lambda_{i}r_{0i}\\ v_{hi}^{*}\left(t+\frac{\mathrm{\Delta t}}{2}\right)\to v_{hi}^{*}\left(t+\frac{\mathrm{\Delta t}}{2}\right)+\frac{1}{2m_{H}}\frac{1}{\mathrm{\Delta t}}\left(\lambda_{i}r_{0i}\right) \end{array}$$

Following the initial coordinate and half‐step velocity correction, the velocity Verlet algorithm evaluates the forces at the constrained coordinates and advances the velocities to the full step, $v_{i}^{*}\left(t+∆t\right)$. A second constraint step is then performed to ensure that the updated velocities satisfy the velocity constraints associated with the constrained bond geometry. Unlike the coordinate constraint step, which is formulated using the unconstrained bond vectors $r_{0i}^{*}$, the velocity correction uses the constrained bond vectors $r_{0i}$. Consequently, the velocity constraint matrix $A_{XH_{n}}^{\mathrm{vel}}$ has the same structure as the coordinate constraint matrix given in Equations (4) and (5), with $r_{0i}^{*}$ simply replaced by $r_{0i}$.

The Lagrange multipliers for the velocity correction are obtained by inverting this matrix and multiplying it by the vector of velocity deviations along the bonds:

(8) $$\left[\begin{array}{c} \lambda_{1}\\ \vdots \\ \lambda_{n} \end{array}\right]={\left(A_{XH_{n}}^{\mathrm{vel}}\right)}^{-1}\left[\begin{array}{c} r_{01}\cdot v_{01}^{*}\\ \vdots \\ r_{0n}\cdot v_{0n}^{*} \end{array}\right]$$

where $v_{0i}^{*}=v_{0}^{*}\left(t+∆t\right)-v_{\mathrm{hi}}^{*}\left(t+∆t\right)$. The resulting Lagrange multipliers are then used to remove velocity components along the constrained bonds while preserving the constrained coordinates obtained in the first correction step. Although both constraint steps employ the same mathematical framework, they serve different purposes: the first enforces the bond‐length constraints on the coordinates, whereas the second ensures that the updated velocities remain consistent with the constrained coordinates.

#### 2.2.2 Constraint Tolerance and Error Propagation

The numerical accuracy of holonomic constraints is controlled by the constraint tolerance adopted in each MD package. Default constraint settings vary considerably across widely used simulation packages [9, 10, 11, 12, 13, 14, 15, 16]. For example, GROMACS employs a relative tolerance of 10^−4^ for SHAKE, whereas LINCS controls constraint accuracy through iterative expansion parameters rather than an explicit distance tolerance [9]. Similarly, default settings in AMBER [13], OpenMM [14], and LAMMPS [15] are generally compatible with FP32 arithmetic. In contrast, GENESIS adopts a strict absolute tolerance of 10^−10^ Å for SHAKE‐type constraints [10, 11, 12]. NAMD has the tolerance value of 10^−8^ Å [16]. CHARMM distinguishes between arithmetic precisions by employing a relative tolerance of 10^−10^ in double‐precision and 10^−5^ in single‐precision [17, 18]. Because these packages use different definitions of constraint tolerance, these values should not be interpreted as directly equivalent. In most MD implementations, discussions of numerical precision have primarily focused on satisfying the coordinate constraint tolerance. However, the choice of tolerance and arithmetic precision has a far more profound impact on velocity accuracy. During the constraint process, the velocity update is obtained by dividing the coordinate correction by the integration time step (Δt). Consequently, a seemingly small coordinate error can be significantly amplified in the velocity update. These velocity‐update errors directly compromise energy conservation, leading to significant energy drift in microcanonical (NVE) simulations.

While standard FP32 arithmetic might initially appear sufficient for basic coordinate tolerances, it struggles to maintain the stringent velocity accuracy required for stable long‐term energy conservation. Conversely, while FP64 natively provides the required accuracy, relying purely on FP64 creates a severe performance bottleneck on modern GPUs—particularly those with limited FP64 throughput. This inherent conflict highlights the necessity for alternative strategies, such as the mixed‐precision and pairwise arithmetic for single‐precision approaches introduced in Section 2.3, which aim to preserve strict tolerance and velocity accuracy without incurring the full computational cost of FP64 arithmetic.

### 2.3 New Implementation of Constraints

#### 2.3.1 Analytical Treatment of One‐Hydrogen Groups ($XH_{1}$)

The first contribution of this work is an analytical treatment of constraints for $XH_{1}$ group (a single hydrogen bonded to a heavy atom). As a practical implementation choice within our M‐SHAKE framework, we employ an exact analytical treatment for the ${\mathrm{XH}}_{1}$ group. Recognized as a known special case for isolated bonds, this eliminates the iterative solution required by the standard SHAKE procedure. The holonomic constraint condition leads to a quadratic equation for the Lagrange multiplier λ:

(9) $$a\lambda^{2}+\mathrm{b\lambda}+c=0$$

with coefficients:

(10) $$\begin{array}{l} a={\left(\frac{1}{m_{X}}+\frac{1}{m_{H}}\right)}^{2}\frac{{\left|r_{01}\left(t\right)\right|}^{2}}{4}\\ b=-\left(\frac{1}{m_{X}}+\frac{1}{m_{H}}\right)r_{01}^{*}\left(t+\mathrm{\Delta t}\right)\cdot r_{01}\left(t\right)\\ c={\left|r_{01}^{*}\left(t+\mathrm{\Delta t}\right)\right|}^{2}-d_{01}^{2} \end{array}$$

We select the root for which λ→0 as c→0, ensuring that the constraint correction vanishes when the predicted bond length approaches the target bond length. In the present implementation, this root is evaluated directly using the conventional quadratic formula rather than a rationalized form. Although direct evaluation can in principle be susceptible to cancelation when c is very small, the accuracy tests presented in Section 3 show that the resulting numerical accuracy is sufficient for the $XH_{1}$ constraints considered here. Because the constraint is solved analytically in a single step, no additional rounding errors accumulate through iterative constraint corrections.

#### 2.3.2 Mixed‐Precision Floating Point Arithmetic for $XH_{2}$ and $XH_{3}$ Groups

For the remaining $XH_{2}$ and $XH_{3}$ groups, analytical treatment is no longer available, and the coupled constraint equations require an iterative numerical solution. To balance computational efficiency with the need to suppress the velocity‐update errors discussed above, we adopt a MP implementation strategy for these groups. As detailed in Algorithm 1, FP64 arithmetic is restricted to the operations most susceptible to rounding errors. Specifically, the predicted bond vectors $r_{0i}^{*}=r_{0}^{*}\left(t+∆t\right)-r_{\mathrm{hi}}^{*}\left(t+∆t\right)$ and the subsequent coordinate updates are evaluated in FP64 because these quantities directly determine the coordinate corrections that propagate into the velocity update. All remaining operations—including the construction of the constraint matrix, matrix inversion, and evaluation of Lagrange multipliers—are performed in FP32. This selective precision strategy minimizes the use of expensive FP64 arithmetic while preserving sufficient numerical accuracy to suppress the amplified velocity‐update errors.

#### 2.3.3 Pairwise Arithmetic Operation (PW‐SP)

Building on the mixed‐precision analysis above, we further reduce the use of FP64 arithmetic in the $XH_{2}$ and $XH_{3}$ constraint calculations by implementing a pairwise arithmetic with single‐precision (PW‐SP) strategy. Pairwise arithmetic represents an extended‐precision number as the unevaluated sum of two FP32 values, as outlined in Algorithm 2. As discussed in the previous section, extended precision is required only for the coordinate accumulation and bond‐vector evaluation steps that are most susceptible to rounding errors. The PW‐SP strategy therefore replaces these specific FP64 operations while leaving the remaining parts of the constraint algorithm unchanged. By applying Algorithm 2 (and its subtraction equivalent, $a-b=a+\left(-b\right)$) exclusively to evaluate the bond vectors ($r_{0i}^{*}$) and accumulate the final coordinate updates, the proposed implementation eliminates the need for native FP64 arithmetic in these operations. This preserves the numerical accuracy required for strict constraint tolerances while improving computational throughput on architectures with limited FP64 performance.

## 3 Results and Discussion

In this section, we evaluate the proposed implementation strategies in terms of numerical accuracy, long‐term energy conservation, and computational performance. We examine the impact of floating‐point precision on velocity accuracy and its consequences for energy conservation. To assess these aspects, we implemented four constraint variants: (1) Double‐precision (DP), where all operations are in FP64; (2) Single‐precision (SP), using only FP32 operations; (3) Mixed‐precision (MP), where temporary coordinate updates are performed in FP64 while all remaining operations use FP32; and (4) Pairwise arithmetic with single‐precision (PW‐SP), which replaces the FP64 bond‐vector evaluation and coordinate updates in the MP scheme with an unevaluated sum of two FP32 numbers.

The benchmark was designed to evaluate each of the three implementation strategies introduced in Section 2.3: the analytical treatment for $XH_{1}$ groups, the MP implementation for $XH_{2}$ and $XH_{3}$ groups, and the PW‐SP implementation. Specifically, a 1,2‐dipalmitoyl‐sn‐phosphatidylcholine (DPPC) bilayer system was employed as the benchmark model. The system consists of 40 lipids in each leaflet (80 lipids in total) and 2701 water molecules, yielding a total of 18,503 atoms. The system contains 160 $XH_{1}$, 5120 $XH_{2}$, and 180 $XH_{3}$ groups, corresponding to a total of 10,940 constrained bonds. This configuration provides a realistic environment with a high density of diverse hydrogen‐constrained groups. Rather than evaluating the constraint solver at a single tolerance level, we systematically varied the number of M‐SHAKE iterations to investigate the relationship between accuracy, energy conservation, and computational cost for the four precision implementations.

### 3.1 Computational Environment

Accuracy tests were performed using both GNU Fortran (GFortran version 8.5) and Intel Fortran (version 2021.9). Consistent results were obtained with both compilers.

Performance benchmarks were conducted on an Intel CPU, an NVIDIA H100 GPU, and two GPUs with limited FP64 throughput (RTX 3080, RTX A6000 Ada). The CUDA kernels were compiled with the options ‐O3 –use_fast_math ‐Xptxas ‐Ofast‐compile=0 ‐dopt=on ‐no‐exceptions ‐extra‐device‐vectorization ‐restrict ‐lineinfo. The architecture‐specific options were ‐gencode arch=compute_86,code = sm_86 for RTX 3080 and RTX A6000 Ada and ‐gencode arch = compute_90,code = sm_90 for the H100. Each CUDA thread processed one $XH_{n}$ group, and the kernels were launched using 190 blocks with 32 threads per block. The CPU benchmark was compiled using Intel Fortran with ‐O3 ‐xHost.

Except for the energy conservation tests, SHAKE and M‐SHAKE were compared using standalone kernel programs. Reported timings include only GPU kernel execution time, excluding host‐device data transfers and synchronization overhead. GPU kernel timings were measured using nsys. The total execution time for 1000 kernel launches was measured and divided to obtain the average execution time. No warm‐up kernel launches were performed before timing.

Although different CUDA Toolkit versions (12.2 for RTX 3080, 12.8 for RTX A6000 Ada, and 12.3 for H100) were used for different GPU platforms, all performance comparisons among the precision implementations were made on the same hardware platform. Therefore, differences in CUDA Toolkit versions do not affect the conclusions regarding the relative performance.

Energy conservation was evaluated using two MD packages. GENESIS was compiled with Intel Fortran compiler (version 2021.9) and the CUDA Toolkit version 12.1, whereas GROMACS was built with GNU C compiler (gcc version 13.0) and the CUDA Toolkit version 12.8. For these tests, the LINCS/SETTLE constraint scheme in GROMACS was compared with SHAKE or M‐SHAKE implementations in GENESIS. Because these packages employ different implementations and compilation environments, the GROMACS results are presented only as qualitative reference points rather than for direct quantitative comparison.

### 3.2 Accuracy Tests of Constraint Schemes

We evaluated constraint accuracy within the standalone constraint kernels using the following two metrics:

(11) $$\begin{array}{l} \varepsilon_{R}=\max_{k}\left(\left|r_{k_{1}k_{2}}-d_{k}\right|\right)\\ \varepsilon_{V}=\max_{k_{1}}\left(\left|v_{k_{1}}-v_{k_{1}}^{\mathrm{ref}}\right|\right) \end{array}$$

where k is the bond index, and $k_{1}$ and $k_{2}$ are the atom indices involved in the bond. These metrics represent the maximum deviation from the target distance and maximum deviation from reference velocity, respectively. Here, $v_{k_{1}}^{\mathrm{ref}}$ is the reference velocity obtained from a fully FP64 (double‐precision) simulation using six M‐SHAKE iterations. Even when running constraint calculations with SP or MP, the accuracy metrics are evaluated after converting all relevant variables to FP64 to ensure a fair comparison. The accuracy was evaluated independently for each $XH_{n}$ group (n=1,2,3). As discussed in Section 2.3, the central focus of these tests is to demonstrate that while the analytical treatment of $XH_{1}$ groups is sufficiently accurate even in pure SP, the $XH_{2}$ and $XH_{3}$ groups suffer from significant velocity‐update errors in pure SP. In the following sections, we show that these amplified errors can be effectively mitigated by employing the MP or PW‐SP approaches.

#### 3.2.1 SHAKE

Using full DP arithmetic, both $\varepsilon_{R}$ and $\varepsilon_{V}$ can be reduced to less than 10^−14^ and 10^−12^, respectively, with six SHAKE iterations. Five iterations are typically sufficient to meet the stringent tolerance thresholds required in GENESIS (see Table 1). However, with pure SP arithmetic, $\varepsilon_{R}$ cannot be reduced below 10^−6^, even with an increased number of iterations. Similarly, $\varepsilon_{V}$ remains above 10^−4^. From the fifth iteration onward, increasing the number of iterations in SP does not improve accuracy and even slightly degrades velocity accuracy. This behavior is consistent with the accumulation of rounding errors inherent in FP32 arithmetic.

**TABLE 1:** $\varepsilon_{R}$ (Å) and $\varepsilon_{V}$ (Å/ps^a^) with SHAKE.

Precision	Iteration	$XH_{1}$		$XH_{2}$		$XH_{3}$	
		$\varepsilon_{R}$	$\varepsilon_{V}$	$\varepsilon_{R}$	$\varepsilon_{V}$	$\varepsilon_{R}$	$\varepsilon_{V}$
DP	1	4.45 × 10^−5^	1.00 × 10^−3^	3.64 × 10^−4^	8.26 × 10^−3^	6.07 × 10^−4^	1.39 × 10^−2^
	2	8.92 × 10^−10^	2.01 × 10^−8^	2.16 × 10^−6^	4.87 × 10^−5^	1.20 × 10^−5^	2.74 × 10^−4^
	3	4.88 × 10^−15^	1.73 × 10^−13^	2.57 × 10^−9^	5.82 × 10^−8^	9.08 × 10^−8^	2.06 × 10^−6^
	4	2.89 × 10^−15^	2.03 × 10^−13^	4.38 × 10^−12^	9.89 × 10^−11^	4.20 × 10^−10^	9.54 × 10^−9^
	5	2.89 × 10^−15^	2.02 × 10^−13^	9.77 × 10^−15^	6.33 × 10^−13^	2.56 × 10^−12^	5.84 × 10^−11^
	6	2.89 × 10^−15^	2.22 × 10^−13^	5.10 × 10^−15^	6.24 × 10^−13^	1.64 × 10^−14^	7.34 × 10^−13^
SP	1	4.33 × 10^−5^	1.00 × 10^−3^	3.64 × 10^−4^	8.27 × 10^−3^	6.06 × 10^−4^	1.39 × 10^−2^
	2	2.24 × 10^−6^	4.57 × 10^−5^	4.00 × 10^−6^	1.11 × 10^−4^	1.23 × 10^−5^	2.91 × 10^−4^
	3	1.89 × 10^−6^	8.50 × 10^−5^	2.57 × 10^−6^	1.73 × 10^−4^	2.13 × 10^−6^	1.58 × 10^−4^
	4	1.86 × 10^−6^	1.27 × 10^−4^	2.57 × 10^−6^	2.02 × 10^−4^	2.13 × 10^−6^	1.83 × 10^−4^
	5	1.86 × 10^−6^	1.70 × 10^−4^	2.57 × 10^−6^	2.49 × 10^−4^	2.13 × 10^−6^	2.23 × 10^−4^
	6	1.86 × 10^−6^	2.12 × 10^−4^	2.57 × 10^−6^	2.95 × 10^−4^	2.13 × 10^−6^	2.61 × 10^−4^

Abbreviations: DP: double‐precision, SP: single‐precision.

^a^ Time is converted to internal units.

#### 3.2.2 M‐SHAKE

Accuracy test results for M‐SHAKE are summarized in Table 2. The DP implementation of M‐SHAKE algorithm demonstrates significantly faster convergence than SHAKE. Even with very tight tolerance values (e.g., < 10^−10^), only three iterations are typically required for convergence. With just one M‐SHAKE iteration, the results are largely indistinguishable across all precision types. However, from the second iteration onward, clear differences emerge. The behavior of $XH_{1}$ and ${\mathrm{XH}}_{2}$/$XH_{3}$ groups is discussed separately below because different implementation strategies are applied.

**TABLE 2:** $\varepsilon_{R}$ (Å) and $\varepsilon_{V}$ (Å/ps^a^) with M‐SHAKE.

Precision	Iteration	$XH_{1}$		$XH_{2}$		$XH_{3}$	
		$\varepsilon_{R}$	$\varepsilon_{V}$	$\varepsilon_{R}$	$\varepsilon_{V}$	$\varepsilon_{R}$	$\varepsilon_{V}$
DP	1	5.11 × 10^−15^	0.00	1.14 × 10^−4^	2.61 × 10^−3^	1.52 × 10^−4^	3.45 × 10^−3^
	2	—	—	6.16 × 10^−9^	1.40 × 10^−7^	1.08 × 10^−8^	2.44 × 10^−7^
	3	—	—	6.88 × 10^−15^	2.84 × 10^−13^	6.00 × 10^−15^	2.77 × 10^−13^
SP	1	1.86 × 10^−6^	3.17 × 10^−6^	1.15 × 10^−4^	2.61 × 10^−3^	1.52 × 10^−4^	3.45 × 10^−3^
	2	—	—	3.10 × 10^−6^	8.15 × 10^−5^	2.87 × 10^−6^	6.57 × 10^−5^
	3	—	—	2.21 × 10^−6^	9.38 × 10^−5^	2.13 × 10^−6^	9.38 × 10^−5^
MP	1	1.86 × 10^−6^	3.17 × 10^−6^	1.15 × 10^−4^	2.61 × 10^−3^	1.52 × 10^−4^	3.45 × 10^−3^
	2	—	—	3.45 × 10^−6^	4.00 × 10^−6^	3.19 × 10^−6^	3.70 × 10^−6^
	3	—	—	3.45 × 10^−6^	3.92 × 10^−6^	3.20 × 10^−6^	3.85 × 10^−6^
PW‐SP	1	1.86 × 10^−6^	3.17 × 10^−6^	1.15 × 10^−4^	2.61 × 10^−3^	1.52 × 10^−4^	3.45 × 10^−3^
	2	—	—	3.45 × 10^−6^	4.00 × 10^−6^	3.19 × 10^−6^	3.67 × 10^−6^
	3	—	—	3.45 × 10^−6^	3.91 × 10^−6^	3.20 × 10^−6^	3.83 × 10^−6^

Abbreviations: DP: double‐precision, MP: mixed‐precision, PW‐SP: pairwise arithmetic with single‐precision, SP: single‐precision.

^a^ Time is converted to internal units.

For $XH_{1}$ groups, there is no observable difference between SP and MP results. As theoretically established in Section 2.3.1, this is because the Lagrange multiplier for $XH_{1}$ is computed analytically, requiring no further iterations. Thus, SP arithmetic is therefore sufficient for $XH_{1}$ groups, as it avoids iterative error accumulation.

For $XH_{2}$ and $XH_{3}$ groups, while coordinate accuracy ($\varepsilon_{R}$) remains similar between SP and MP, the precision strategy significantly impacts velocities. The MP approach substantially reduces velocity‐update errors relative to pure SP, achieving roughly one order of magnitude better velocity accuracy. In MP, the accuracy of velocities closely matches that of the coordinates, indicating that our selective FP64 usage successfully prevents error accumulation, indicating that the dominant source of errors is limited to the FP32 coordinate prediction rather than the constraint correction itself. In contrast, the purely SP implementation exhibits velocity accuracy that is consistently lower than coordinate accuracy, indicating that rounding errors are amplified during the velocity correction phase. Like standard SHAKE, increasing the number of iterations beyond two in purely SP M‐SHAKE leads to a degradation in velocity accuracy due to cumulative rounding errors.

Replacing the critical FP64 operations with the PW‐SP strategy substantially reduces the velocity‐update errors relative to the pure SP implementation. The resulting accuracy is comparable to that of the MP implementation while avoiding the use of native FP64 arithmetic.

### 3.3 Energy Drift

To evaluate the energy conservation performance of the proposed precision strategies, we monitored the energy drift in the microcanonical (NVE) ensemble. To distinguish the effects of integration precision from those of the constraint implementation, seven simulation setups were considered.

-
  Case 1 DP for both integration and constraints (RATTLE for $XH_{n}$ groups, SETTLE for water molecules)
-
  Case 2 DP for both integration and constraints (M‐SHAKE for all constraints, including water molecules)
-
  Case 3 SP for integration and DP for constraints (RATTLE for $XH_{n}$ groups, SETTLE for water molecules)
-
  Case 4 SP for integration and MP for constraints (M‐SHAKE for all constraints including water)
-
  Case 5 SP for both integration and constraints (M‐SHAKE for all constraints including water)
-
  Case 6 SP for both integration and constraints, with PW‐SP (M‐SHAKE for all)
-
  Case 7 GROMACS reference (DP, version 2025.0, LINCS with one and four iterations, Verlet‐buffer‐tolerance equivalent to 10^−8^, SETTLE for water)

For Cases 1 through 6, all NVE simulations were performed using the velocity Verlet integrator in GENESIS. Cases 4, 5, 6 are designed to isolate the influence of the constraint implementation while keeping the integration precision unchanged. For Case 7, the simulations were performed using the md integrator in GROMACS. In Cases 1 and 3, the constraint tolerance was set to 10^−10^ Å for $XH_{n}$ groups.

**FIGURE 1:**
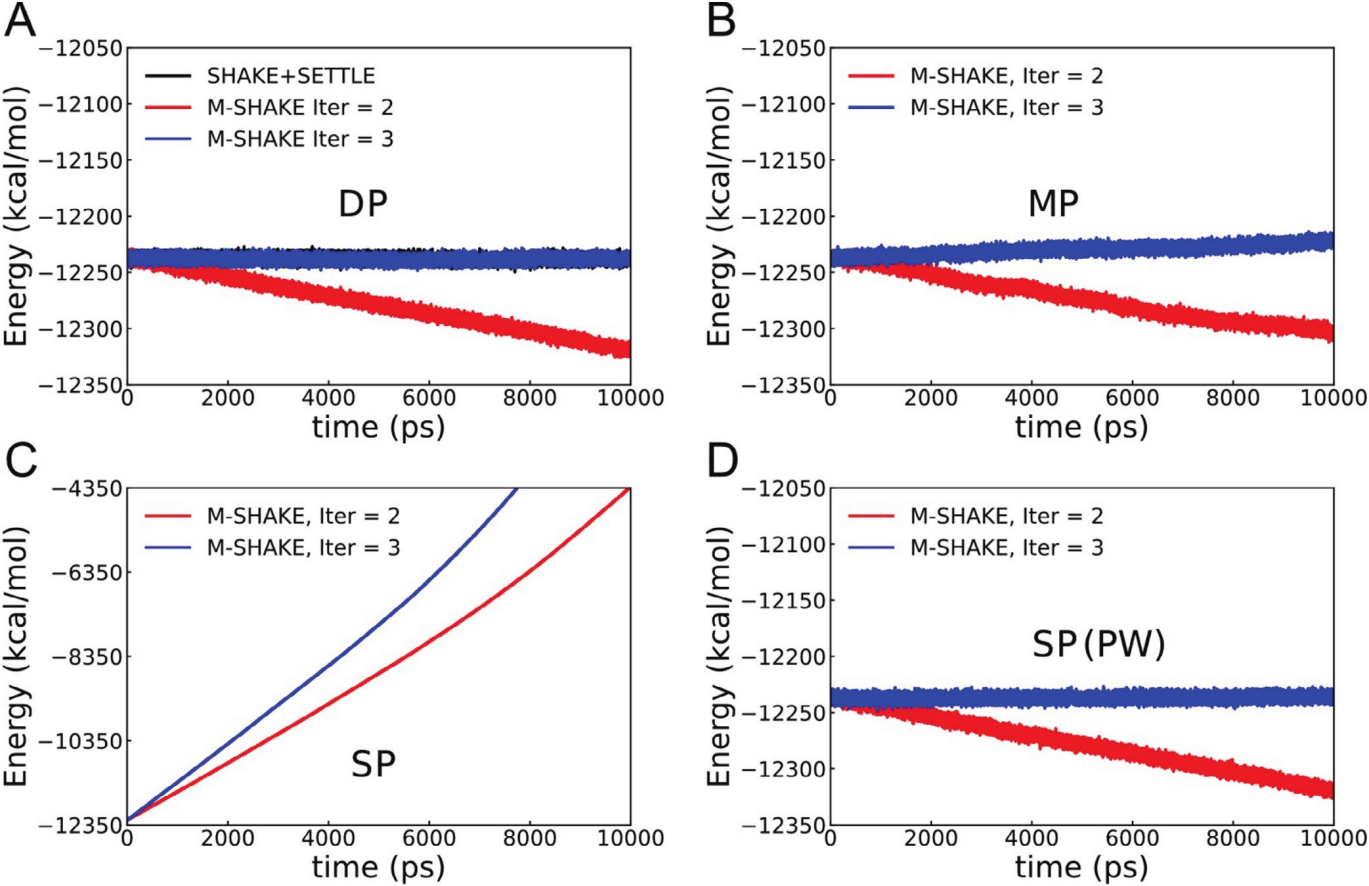
Total energy versus time in the microcanonical ensemble simulation. (A) DP: double‐precision for integration and constraints (Case 1 and Case 2). (B) SP: single‐precision for integration and mixed‐precision for constraints (Case 4). (C) Single‐precision for integration and constraints (Case 5). (D) SP (PW): single‐precision for integration and constraints where constraints pairwise arithmetic operation is applied to reduce the round error from single‐precision floating‐point.

The total energy over time is plotted in Figure 1, and the energy drift, computed as the slope of the linear regression line, is quantified in Table 3. Cases 1 and 2, where both the integrator and the constraint are calculated in DP, show excellent energy conservation, with negligible drift (below 10^−8^ kcal/mol/atom/ps) over the entire 10 ns. Cases 3 and 4 exhibit similar levels of energy drift to each other, indicating that the MP implementation provides energy conservation comparable to that obtained with DP constraints using a tolerance of 10^−10^ and the SETTLE algorithm for water molecules. From the result of Cases 2 and 4, we note that using M‐SHAKE with only two iterations introduces noticeable energy drift. This reflects incomplete convergence of the constraint correction at two iterations. Case 5 (SP only) yields a 500‐fold larger drift than Case 4. This result highlights the importance of suppressing velocity‐update errors through the MP implementation. This observation is consistent with the analysis in Section 2.2, where accumulated rounding errors in the velocity correction were identified as the primary source of long‐term energy drift. Replacing the FP64 operation in Case 4 with PW‐SP strategy preserves the same level of energy conservation when three M‐SHAKE iterations are employed, with no distinguishable difference from the fully DP reference (Case 2).

**TABLE 3:** Energy drift under the NVE condition (unit: kcal/mol/ps/atom).

Case	Precision (integration/constraints)	Constraints of $XH_{n}$	Constraints of water	Constraint condition	Energy drift^a^
1	Double/double	SHAKE	SETTLE	Tol = 10^−10^Å	7.32 × 10^−9^
2	Double/double	M‐SHAKE	M‐SHAKE	Iter = 2	4.42 × 10^−7^
				Iter = 3	3.10 × 10^−9^
3	Single/double	SHAKE	SETTLE	Tol = 10^−10^Å	4.21 × 10^−8^
4	Single/mixed	M‐SHAKE	M‐SHAKE	Iter = 2	3.73 × 10^−7^
				Iter = 3	7.47 × 10^−8^
5	Single/single	M‐SHAKE	M‐SHAKE	Iter = 2	4.17 × 10^−5^
				Iter = 3	5.95 × 10^−5^
6	Single/single (PW)	M‐SHAKE	M‐SHAKE	Iter = 2	4.44 × 10^−7^
				Iter = 3	8.09 × 10^−9^
7	Double/double	LINCS^b^	SETTLE	Iter = 1	4.72 × 10^−4^
				Iter = 4	2.45 × 10^−5^

^a^ We output the absolute value of the energy drift.

^b^ Simulations performed using GROMACS 2025.0.

Finally, GROMACS (Case 7) is provided strictly as a contextual reference. Despite using DP and four LINCS iterations, it shows larger energy drift than our proposed MP method. However, because energy drift across different software stacks is affected by many factors, this difference cannot be uniquely attributed to the precision of the constraint. Nevertheless, the GROMACS results provide additional qualitative evidence that the proposed constraint implementation achieves excellent energy conservation within the GENESIS framework.

### 3.4 GPU Performance of Constraints

**FIGURE 2:**
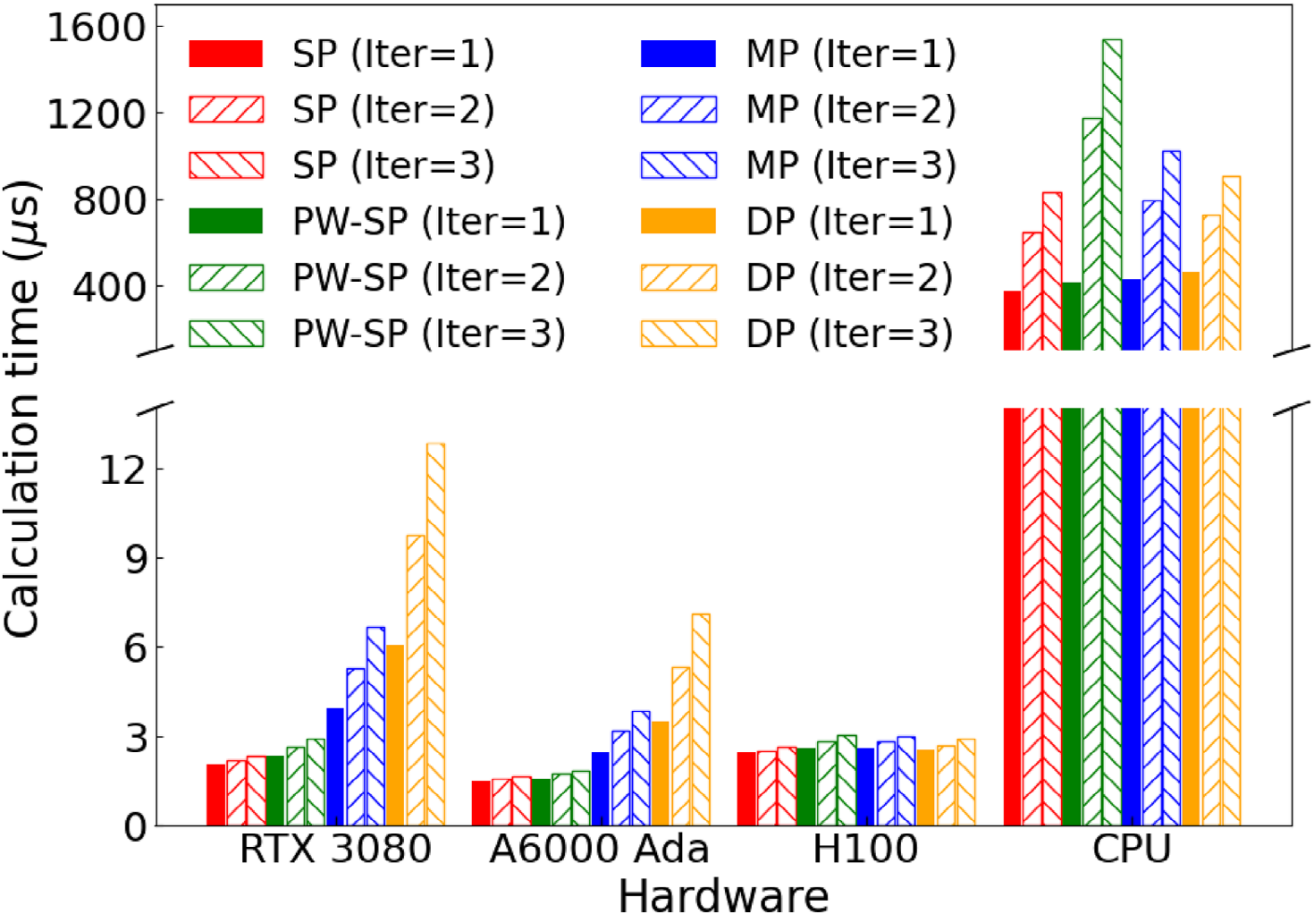
Performance comparison of M‐SHAKE constraints on GPUs and CPU. The vertical axis represents the elapsed time per constraint calculation, and the horizontal axis shows different CPU/GPU configurations. Colors indicate the precision (DP, MP, SP, and PW‐SP), while the bar patterns represent the number of iterations.

Our goal is to enable GPU acceleration while maintaining the accuracy demonstrated in Section 3.3. We implemented standalone CUDA kernels for constraint calculations in DP, MP, and SP, and PW‐SP, and benchmarked them on three GPUs (RTX 3080, A6000 Ada, and H100) and one CPU core (Intel Xeon Silver 4210). Figure 2 presents the performance over different iteration counts, whereas Table 4 summarizes the execution times and relative speedups for the iteration count (three iterations) that provides equivalent numerical accuracy across the DP, MP, and PW‐SP implementations. The comparisons in Table 4 are restricted to iteration counts that provide acceptable numerical accuracy, thereby excluding the pure SP implementation from the performance comparison.

**TABLE 4:** Kernel execution time and relative speedup of the DP, MP, and PW‐SP constraint implementations at equivalent numerical accuracy.

Hardware	Scheme	Iterations^a^	Time (μs)	Speedup versus DP
RTX 3080	DP	3	12.85	1.00
	MP	3	6.66	1.93
	PW‐SP	3	2.88	4.46
A6000 Ada	DP	3	6.99	1.00
	MP	3	3.82	1.83
	PW‐SP	3	1.80	3.89
H100	DP	3	2.89	1.00
	MP	3	2.98	0.97
	PW‐SP	3	3.05	0.95

*Note:* The reported iteration counts apply to $XH_{2}$ and $XH_{3}$ groups. $XH_{1}$ constraints are solved analytically in a single noniterative step using standard single‐precision arithmetic.

^a^ All timings correspond to three M‐SHAKE iterations, which were sufficient to achieve the target numerical accuracy for DP, MP, and PW‐SP implementations (see Section 3.2).

For the RTX 3080 and A6000 Ada, MP was slower by approximately 50–150% compared to SP, and DP was the slowest, being up to five times slower than SP. This is due to the significantly lower throughput of FP64 cores compared to FP32 cores on these architectures. While MP provides a good compromise, a noticeable performance gap remains between SP and MP. By slightly increasing the number of operations using PW‐SP while keeping all calculations in SP, we can substantially reduce the calculation time on these GPUs with limited FP64 throughput, making PW‐SP the fastest accuracy‐preserving variant.

For the H100, which has similar performance for FP32 and FP64 when vectorization is absent, the runtimes for SP and DP were nearly identical. MP was slightly slower than DP, primarily due to the overhead of casting between FP64 and FP32 within the kernel. The pairwise algorithm increased the operation count without outperforming DP. These results suggest that for GPUs with limited FP64 throughput, constraints in SP combined with a pairwise algorithm in error‐sensitive parts offer the best balance between accuracy and performance. Conversely, for HPC‐class GPUs, DP remains preferable for the specific constraint kernels evaluated in this study.

While pairwise arithmetic with SP is an effective solution for GPUs, it is not recommended for CPUs within the context of these constraint solvers. For the Intel Xeon processor used in this study, no significant performance difference was observed between SP and DP. In fact, MP and pairwise‐arithmetic schemes resulted in longer wall‐clock times than standard DP due to the additional operation overhead.

## 4 Conclusion

We presented optimized implementations of the M‐SHAKE constraint algorithm that improve computational performance while maintaining numerical accuracy. Our methodology combines an analytical treatment for $XH_{1}$ groups with precision‐level implementation strategies for iterative constraints. First, for $XH_{1}$ groups, we showed that an analytical treatment intrinsically prevents iteration‐based error accumulation. Consequently, pure single‐precision (SP) arithmetic is sufficient for these constraints, with SP, MP, and PW‐SP yielding essentially the same numerical accuracy. Second, for the iterative constraints required by $XH_{2}$ and $XH_{3}$ groups, we introduced a mixed‐precision (MP) approach and a pairwise single‐precision (PW‐SP) approach. Both methods preserve near‐maximum accuracy at the SP level by significantly reducing velocity‐update errors, which are otherwise prominent in pure SP calculations. Accuracy was evaluated through coordinate and velocity accuracy together with energy drift analysis. The new schemes yielded substantially better energy conservation than pure SP. The improved long‐term energy conservation observed in the NVE simulation is consistent with our analysis that suppressing velocity‐update errors is essential for maintaining numerical stability under reduced‐precision arithmetic. These precision‐level optimizations are effective for improving performance on GPUs with limited FP64 throughput. In particular, the PW‐SP approach achieved computational execution times comparable to pure SP, while simultaneously maintaining the improved accuracy required for long‐term stability.

Overall, our results demonstrate that combining the analytical treatment for $XH_{1}$ groups with the MP or PW‐SP strategies for iterative constraints provides an effective balance between numerical accuracy and computational performance on GPUs with limited FP64 throughput. Conversely, conventional double‐precision remains preferable for HPC‐class GPUs with high FP64 throughput for the specific standalone constraint kernels evaluated in this study. These conclusions are based on benchmarks using a representative DPPC lipid bilayer system and standalone constraint‐kernel measurements, and further validation using additional biomolecular systems and full application‐level benchmarks will be valuable in future work.

## Funding

This work was supported by the Ministry of Education, Culture, Sports, Science and Technology (19H05645, 21H05249, 21H05282, 23K11328).

## Data Availability

The data that support the findings of this study are available from the corresponding author upon reasonable request.

## References

[1] C. L. Brooks III , A. D. Mackerell Jr , C. B. Post , and L. Nilsson , “Biomolecular Dynamics in the 21st Century,” BBA‐General Subjects 1868, no. 2 (2024): 130534. 10.1016/j.bbagen.2023.130534 PMC10842176 38065235

[2] J. P. Ryckaert , G. Ciccotti , and H. J. C. Berendsen , “Numerical‐Integration of Cartesian Equations of Motion of a System with Constraints ‐ Molecular‐Dynamics of N‐Alkanes,” Journal of Computational Physics 23, no. 3 (1977): 327–341.

[3] H. C. Andersen , “Rattle ‐ A Velocity Version of the Shake Algorithm for Molecular‐Dynamics Calculations,” Journal of Computational Physics 52, no. 1 (1983): 24–34.

[4] S. Miyamoto and P. A. Kollman , “Settle ‐ An Analytical Version of the Shake and Rattle Algorithm for Rigid Water Models,” Journal of Computational Chemistry 13, no. 8 (1992): 952–962.

[5] B. Hess , H. Bekker , H. J. C. Berendsen , and J. G. E. M. Fraaije , “LINCS: A linear Constraint Solver for Molecular Simulations,” Journal of Computational Chemistry 18, no. 12 (1997): 1463–1472.

[6] V. Kräutler , W. F. Van Gunsteren , and P. H. Hünenberger , “A Fast SHAKE Algorithm to Solve Distance Constraint Equations for Small Molecules in Molecular Dynamics Simulations,” Journal of Computational Chemistry 22, no. 5 (2001): 501–508.

[7] M. Lange and S. M. Rump , “Faithfully Rounded Floating‐point Computations,” ACM Transactions on Mathematical Software 46, no. 3 (2020): 1–20.

[8] W. C. Swope , H. C. Andersen , P. H. Berens , and K. R. Wilson , “A Computer‐Simulation Method for the Calculation of Equilibrium‐Constants for the Formation of Physical Clusters of Molecules ‐ Application to Small Water Clusters,” Journal of Chemical Physics 76, no. 1 (1982): 637–649.

[9] S. Páll , A. Zhmurov , P. Bauer , et al., “Heterogeneous Parallelization and Acceleration of Molecular Dynamics Simulations in GROMACS,” Journal of Chemical Physics 153, no. 13 (2020): 134110. 10.1063/5.0018516 33032406

[10] J. Jung , T. Mori , C. Kobayashi , et al., “GENESIS: A Hybrid‐parallel and Multi‐scale Molecular Dynamics Simulator with Enhanced Sampling Algorithms for Biomolecular and Cellular Simulations,” Wiley Interdisciplinary Reviews: Computational Molecular Science 5, no. 4 (2015): 310–323. 10.1002/wcms.1220 PMC4696414 26753008

[11] C. Kobayashi , J. Jung , Y. Matsunaga , et al., “GENESIS 1.1: A Hybrid‐parallel Molecular Dynamics Simulator with Enhanced Sampling Algorithms on Multiple Computational Platforms,” Journal of Computational Chemistry 38, no. 25 (2017): 2193–2206. 10.1002/jcc.24874 28718930

[12] J. Jung , K. Yagi , C. Tan , et al., “GENESIS 2.1: High‐Performance Molecular Dynamics Software for Enhanced Sampling and Free‐Energy Calculations for Atomistic, Coarse‐Grained, and Quantum Mechanics/Molecular Mechanics Models,” Journal of Physical Chemistry B 128, no. 25 (2024): 6028–6048. 10.1021/acs.jpcb.4c02096 PMC11215777 38876465

[13] D. A. Case , T. E. Cheatham, 3rd , T. Darden , et al., “The Amber Biomolecular Simulation Programs,” Journal of Computational Chemistry 26, no. 16 (2005): 1668–1688. 10.1002/jcc.20290 PMC1989667 16200636

[14] P. Eastman , J. Swails , J. D. Chodera , et al., “OpenMM 7: Rapid Development of High Performance Algorithms for Molecular Dynamics,” PLoS Computational Biology 13, no. 7 (2017). 10.1371/journal.pcbi.1005659 PMC5549999 28746339

[15] A. P. Thompson , H. M. Aktulga , R. Berger , et al., “LAMMPS ‐ A Flexible Simulation Tool for Particle‐based Materials Modeling at the Atomic, Meso, and Continuum Scales,” Journal of Computational Physics in Communications 271 (2022).

[16] J. C. Phillips , D. J. Hardy , J. D. C. Maia , et al., “Scalable Molecular Dynamics on CPU and GPU Architectures with NAMD,” Journal of Chemical Physics 153, no. 4 (2020): 044130. 10.1063/5.0014475 PMC7395834 32752662

[17] W. Hwang , S. L. Austin , A. Blondel , et al., “CHARMM at 45: Enhancements in Accessibility, Functionality, and Speed,” Journal of Physical Chemistry B 128, no. 41 (2024): 9976–10042. 10.1021/acs.jpcb.4c04100 PMC11492285 39303207

[18] CHARMM , https://academiccharmm.org/documentation/version/c44b2/cons.

